# Report to the Surgeon General on the Minute Anatomy of Two Cases of Cancer

**Published:** 1872-08

**Authors:** 


*Report to the Surgeon General of the United States Army on the Minute Anatomy of two cases of Cancer.* By Assistant Surgeon J. J. WOODWARD, U. S. A.

The usual methods of representing microscopical preparations are open to many objections, the chief one being their want of accuracy. The cost of photographic plates, where a large number of representations are to be made, is an objection against that process which carries much weight with it. The need of some method to overcome these objections, seems to be met by the recent introduction in the United States of a method of printing in ink from photographic negatives. There are at present two methods in operation, the Woodbury and the Albertype processes.

The illustrations in this report, two in number, are made by the Albertype process. We quote from the author a brief description of this method: "In this process a printing surface (not a relief) is produced on a gelatin film by the action of light through the negative, on certain chemicals contained in the film. The surface thus produced when properly inked, yields in the press an impression on paper, in which the details of the original negative are very well preserved. The prints may be made on either plain or enameled paper; in the latter case they closely resemble silver prints on albumen paper, in the former they are like silver prints on plain paper."

We are very much pleased with the illustrations in this paper, and are inclined to favor the print on plain paper; the glare from the surface of the enameled paper having a tendency to obscure the print in certain lights.

The report is very well made, and will add much of value to that which is already known in the minute anatomy of cancer. Case I is a report of a case of mammary cancer. No. II being the report of a case of cancer of the liver. Too much cannot be said in praise of Surgeon Woodward's researches in microscopic anatomy.

